# A Sigmoid Functional Response Emerges When Cytotoxic T Lymphocytes Start Killing Fresh Target Cells

**DOI:** 10.1016/j.bpj.2017.02.008

**Published:** 2017-03-28

**Authors:** Saikrishna Gadhamsetty, Athanasius F.M. Marée, Joost B. Beltman, Rob J. de Boer

**Affiliations:** 1 Theoretical Biology, Utrecht University, Utrecht, the Netherlands; 2 Division of Toxicology, Leiden Academic Centre for Drug Research, Leiden University, Leiden, the Netherlands; 3 Department of Computational and Systems Biology, John Innes Centre, Norwich, United Kingdom

## Abstract

Cytotoxic T lymphocyte (CTL)-mediated killing involves the formation of a synapse with a target cell, followed by delivery of perforin and granzymes. Previously, we derived a general functional response for CTL killing while considering that CTLs form stable synapses (i.e., single-stage) and that the number of conjugates remains at steady state. However, the killing of target cells sometimes requires multiple engagements (i.e., multistage). To study how multistage killing and a lack of steady state influence the functional response, we here analyze a set of differential equations as well as simulations employing the cellular Potts model, in both cases describing CTLs that kill target cells. We find that at steady state the total killing rate (i.e., the number of target cells killed by all CTLs) is well described by the previously derived double saturation function. Compared to single-stage killing, the total killing rate during multistage killing saturates at higher CTL and target cell densities. Importantly, when the killing is measured before the steady state is approached, a qualitatively different functional response emerges for two reasons: First, the killing signal of each CTL gets diluted over several targets and because this dilution effect is strongest at high target cell densities; this can result in a peak in the dependence of the total killing rate on the target cell density. Second, the total killing rate exhibits a sigmoid dependence on the CTL density when killing is a multistage process, because it takes typically more than one CTL to kill a target. In conclusion, a sigmoid dependence of the killing rate on the CTLs during initial phases of killing may be indicative of a multistage killing process. Observation of a sigmoid functional response may thus arise from a dilution effect and is not necessarily due to cooperative behavior of the CTLs.

## Introduction

Cytotoxic T lymphocyte (CTL)-mediated killing of tumor and virus-infected cells generally involves four steps: localization of the target cell; formation of a specialized junction with the target (called a “cytotoxic synapse”); delivery of effector molecules, such as perforin and granzymes; and detachment from the dying target, followed by resumption of the search for new targets. The functional response of CTL-mediated killing is defined as the rate at which a single CTL kills target cells as a function of the CTL and target cell frequencies, and has been studied using mathematical models that are analogous to enzyme-substrate kinetics (1, 2, 3, 4). In such models, the conjugates (i.e., CTLs and target cells that are bound by a synapse between them) either dissociate prematurely resulting in a naïve target cell, or proceed to target cell death. Thus, targets were assumed to be killed after a single cytotoxic synapse during which a lethal hit is delivered.

However, recent in vivo experiments using intravital two-photon microscopy revealed that virus-infected cells break their synapses with CTLs, and tend to be killed during subsequent conjugates with other CTLs (5). In these experiments, CTLs rarely formed stable synapses and remained motile after contacting a target cell. The probability of death of infected cells increased for targets contacted by more than two CTLs, which was interpreted as evidence for CTL cooperation (5). Similarly, with in vitro collagen gel experiments, ∼50% of the HIV-infected CD4^+^ T cells remained motile and broke their synapses with CD8^+^ T cells (6). This study further suggested that the avidity between TCRs and pMHCs plays an important role in the stability of the synapse: an increase in the peptide concentration used for pulsing the target cells, or an increase of the avidity of the peptide, increased the killing efficiency of the first target cell encounter by a CTL (6). In analogy to the short-lived kinapses between T cells and dendritic cells presenting antigen with intermediate or low affinity (7, 8, 9), these short-lived cytotoxic synapses have been called “kinapses” (5). Thus, depending on the antigen concentration and the avidity of the interaction, the killing of a target cell may take several short kinapses (hereafter referred to as “multistage” killing), rather than the one long synapse (hereafter referred to as “single-stage” killing) that was assumed in the modeling hitherto (1, 2, 3, 4).

Additionally, models of CTL-mediated killing typically derive the functional response of CTL-mediated killing by making a quasi-steady-state assumption (QSSA) and consider situations where the number of conjugates remains close to steady state, or changes slowly (1, 2, 4). This assumption is likely to be violated in experiments where fresh target cells and CTLs are mixed, because the first conjugates can only be formed after these cells have found each other. When synapses are long lived, it may take a long time before the number of conjugates in the experiment approaches steady state (4). Moreover, during the acute stage of an infection the number of target cells is increasing, and additional CTLs are arriving from the circulation, which may undergo further clonal expansion. In these examples, it seems unlikely that the total number of conjugates is at (quasi) steady state, and it is unclear how the lack of steady state influences the functional response.

Here, we study how multistage killing and the early killing kinetics before reaching steady state affect the functional response. To this purpose, we adapt our previous simulations in which realistically shaped CTLs and targets migrate and interact in a two-dimensional (2D) environment (4) in two ways. First, the target cells accumulate the killing signal during consecutive short-lived kinapses rather than long-lived synapses with CTLs, until a threshold of killing signal is reached upon which they are killed. Second, we study the effect of the steady state by comparing the killing during the last and the first period of the simulations, representing the scenarios when the killing kinetics are or are not at steady state, respectively. To corroborate the spatial simulation results, we also analyze a set of ordinary differential equations (ODEs) for a well-mixed system.

When the killing is at steady state, our simulations show that the multistage killing only affects the results in a quantitative manner. Thus, the double saturation function, previously identified to describe single-stage killing (4), also well describes the numbers of cells killed observed in multistage killing simulations. For a given killing signal threshold, we find higher saturation constants during multistage than in the single-stage killing simulations—a result that we confirm analytically. In contrast, when the CTL-mediated killing is not at steady state, the functional response exhibits a sigmoid dependence of the killing rate on the CTL density, and the dependence on the target cell density contains a peak. Both modifications to the functional response can mathematically be described by phenomenologically adding exponents to the double saturation function. We conclude that the observed killing rate critically depends on the phase during which measurements are taken, i.e., before or after approaching steady state. Importantly, short-lived cytotoxic synapses are expected to result in a sigmoid dependence of the killing rate on CTL densities that looks like cooperative killing.

## Materials and Methods

### Spatial simulations

To determine the functional response of CTL-mediated killing resulting from multistage killing, we perform 2D cellular Potts model (CPM) simulations (10, 11) that are similar to those in our earlier study (4). The major differences are 1) the stability of the synapse, and 2) the time at which the killing rate is assessed. Briefly, each cell in the CPM is a collection of multiple lattice sites, with surface energies on the edges of a cell determining its interactions with neighbors. We consider a 2D torus of 500 × 500 pixels, where the length of each pixel corresponds to 1 *μ*m, and is composed of static circular elements representing the reticular network in lymph nodes, 2250 CTLs, 2250 B cells as targets, and an extracellular matrix. We perform simulations with different numbers of antigen-bearing target cells and their cognate CTLs, while keeping the total number of CTLs and target cells constant (2250 cells each). After initializing the simulation field with CTLs and target cells at random locations, we let the CTLs and target cells migrate for 40 s. After this burn-in phase, CTLs are allowed to kill target cells. Each CTL and B cell is assigned a preferred direction of migration, which is updated once in every 3 min based on its recent direction of migration, resulting in a self-adjusting motility (12). We consider four scenarios of killing depending on the number of CTLs and target cells in a conjugate: monogamous, joint, simultaneous, and mixed. All the names of these scenarios are chosen from the perspective of CTLs. In monogamous killing, only conjugates of one CTL and one target cell are allowed to form. In joint killing, a target cell can be bound and jointly killed by multiple CTLs but a CTL can only kill a single target cell at a time. Simultaneous killing is the inverse scenario to joint killing, in which a single CTL can simultaneously bind and kill multiple target cells but a target cell can only be killed by one CTL. Finally, in the mixed killing scenario (i.e., a mix of joint and simultaneous scenarios), there are no restrictions and conjugates of multiple CTLs and multiple target cells are allowed to form. In all our simulations, we specify whether more than one CTL or target cell are allowed in a conjugate, but the maximum number of CTLs and target cells in a conjugate emerges in the simulations.

As in our earlier study (4), we mimicked single-stage killing by aiming for stable conjugates through increasing the adhesion upon contact formation (where a contact occurs when a cell has at least one pixel that directly touches the neighboring cell), and by stopping the active migration of cells in conjugates. However, note that in such CPM simulations conjugates still occasionally dissociate due to random membrane fluctuations (13, 14). To simulate multistage killing, we used a low adhesion strength between cells in a conjugate and considered that they continued to migrate independently even when in conjugate (see below for the default parameters used). Together, this results in conjugates of short duration (i.e., cytotoxic kinapses). Note that the adhesion values are chosen empirically to modulate the strength of preference for CTL-target cells to be together in conjugates. Target cells remember the accumulated duration in conjugates after dissociation, and accrue upon this existing signal when a subsequent kinapse is formed with another (or the same) CTL, thus mimicking multistage killing. This memory of the killing signal represents the best-case scenario from the perspective of CTLs as the signal does not decay between consecutive kinapses. Because there is no experimental evidence comparing the times required to induce target cell death involving synapses or kinapses, we here choose this best-case scenario and examine its implications (yet this does not qualitatively affect our results; see Discussion). In our default single- and multistage killing simulations, we used a fixed total killing time of *t*_*D*_ = 15 min, representing the total conjugation time required to induce target cell death. Moreover, we investigate the effect of stochasticity in the killing times by drawing the killing time for new target cells randomly from a Gaussian distribution with mean *μ*_*k*_ and *σ*_*k*_ (requiring the resulting killing time to be positive).

Depending on the killing regime, targets accumulate the killing signal independently from all CTLs with which they share a synapse or kinapse, and CTLs can contribute killing signal to all the targets in their conjugate. For example, a target cell that has a synapse or kinapse with three CTLs at all times is killed three times faster, i.e., requires *t*_*D*_/3 = 5 min. When a killed target disappears from the field, a new target cell is introduced at a random location in the field to maintain the same target cell numbers within a simulation. To prevent creating the new target cells inside another cell, the randomly chosen position is forced to be either within the free extracellular space (which is ∼5% of the field in our simulations), or at the interface between two cell boundaries. The number of CTLs also remains constant within a simulation, thus preventing confounding effects due to changing cell numbers. Each simulation corresponds to 450 min, and to study the initial transient we measure the number of target cells killed over the first 75 min, unless otherwise specified. To study killing at steady state, we measure the number of target cells killed over the last 75 min.

### Default model parameters

The CPM describes the cell behavior and its parameters, which include the surface energies and surface adhesions, have no direct biological meaning, and are chosen such that we approximate the CTL migration properties observed in experiments (15). At each time step, all pixels are considered for extension into a random neighboring site, and one time step in the simulation (i.e., attempting to update all the lattice sites) corresponds to 1 s in real-time. The change in surface energy due to an extension is calculated by the difference in Hamiltonians *H* of two configurations. The Hamiltonian is given by

(1) $$H=\sum_{ij}\sum_{i^{\prime }j^{\prime }}J_{\tau \left(\sigma_{ij}\right),\tau \left(\sigma_{i^{\prime }j^{\prime }}\right)}\left(1-\delta_{\sigma_{ij},\sigma_{i^{\prime }j^{\prime }}}\right)+\sum_{\sigma }\lambda {\left(a_{\sigma }-A_{\tau \left(\sigma \right)}\right)}^{2},$$

where $J_{\tau \left(\sigma_{ij}\right),\tau \left(\sigma_{i^{\prime }j^{\prime }}\right)}$ is the surface energy associated between a lattice site (of state $\sigma_{ij}$ and cell type $\tau \left(\sigma_{ij}\right)$) and the neighboring lattice site (of state $\sigma_{i^{\prime }j^{\prime }}$ and cell type $\tau \left(\sigma_{i^{\prime }j^{\prime }}\right)$), *λ* is the inelasticity, *δ* is the Kronecker delta, $a_{\sigma }$ is the actual area of the cell *σ*, and $A_{\tau \left(\sigma \right)}$ is the target area of cells of type τ(σ). The probability that a lattice site is copied into the neighboring site obeys a Boltzmann equation (i.e., is 1 if Δ*H* < 0, and *e*^−Δ*H*/*D*^ otherwise), where *D* represents the membrane fluctuation amplitude of cells (see Table 1 for the list of parameters used). The surface energies *J* and the surface tensions *γ* are chosen such that the noncognate interactions between any pair of cells are neutral (see Table 2), i.e., there is no preferential adhesion. The entire model is implemented in the C programming language.

### Mathematical models of CTL-mediated killing

For steady-state conditions, we have previously shown that a double saturation (DS) function with two saturation constants (one for CTL and one for target cell densities) describes the CTL-mediated killing rate resulting from all scenarios (4). The DS function is given by

(2) $$K_{\text{DS}}=\Delta t\;\frac{k\bar{E}\bar{T}}{1+\bar{E}/h_{E}+\bar{T}/h_{T}},$$

where Δ*t* is the duration over which killing is measured; $\bar{E}$ and $\bar{T}$ values are the total number of CTLs and target cells, respectively; and *h*_*E*_ and *h*_*T*_ are the Michaelis-Menten saturation constants for CTL and target cell densities. This DS function was mechanistically derived for monogamous and simultaneous killing regimes using a Padé approximation of the full solution.

### Nonlinear regression (or fit) to the data

For all the nonlinear regression analysis of models to the data, we used the functions nlinfit in MATLAB (The MathWorks, Natick, MA) and/or modFit in the flexible modeling environment package of the software environment R (https://www.r-project.org/), implementing the Levenberg-Marquardt algorithm. The 95% confidence intervals are estimated using the nlparci function in MATLAB, which uses an algorithm based on asymptotic normal distributions for the parameter estimates, and/or in R by bootstrapping the data with replacement from 1000 independent samples.

## Results

### Multistage killing at steady state

We start our analysis by studying the differences between short- and long-lived synapses at steady state, i.e., when the functional response can be derived using a QSSA. To this purpose, we analyze a system of ODEs, and perform CPM simulations under steady-state assumptions for the monogamous killing regime. After confirming our results analytically, we consider the three other nonmonogamous killing regimes (joint, simultaneous, and mixed killing; see Materials and Methods for the description of regimes). In all cases, we assess the functional response by measuring the total number of target cells killed after the killing rate has approached steady state.

#### An ODE model for multistage monogamous killing

In monogamous simulations, only conjugates of one CTL and one target are allowed to form. Multistage killing in this case implies that a target cell is killed by multiple CTLs in a sequential manner. To study the effect of multistage killing in a fair manner, the expected time a target in total spends conjugated to CTLs before being killed should be the same for single- and multistage killing (i.e., should be independent of the number of stages). For *n* stages, this is achieved by setting the killing rate to *nk*_2_. The reaction scheme for multistage killing thus becomes

(3) $$\begin{array}{c} E+T_{0}\overset{k_{1}}{\underset{k_{-1}}{⇌}}\left[ET_{1}\right]\overset{nk_{2}}{\to }E+T_{1},\\ E+T_{1}\overset{k_{1}}{\underset{k_{-1}}{⇌}}\left[ET_{2}\right]\overset{nk_{2}}{\to }E+T_{2},\\ \vdots \\ E+T_{n-1}\overset{k_{1}}{\underset{k_{-1}}{⇌}}\left[ET_{n}\right]\overset{nk_{2}}{\to }E+T^{\text{∗}}, \end{array}$$

where *E*, *T*_0_, *T*_1_,…,*T*_*n*−1_, and *T*^∗^ represent free cognate CTLs, naïve target cells, partially lysed targets, and dead targets, respectively. Furthermore, $\left[ET_{1}\right]\ldots \left[ET_{n}\right]$ represent the conjugates during the various stages of killing, *k*_1_ and *k*_−1_ are the rates of conjugate formation and dissociation, and *nk*_2_ is the rate at which targets transit each stage (i.e., *k*_2_ is the killing rate of target cells during single-stage killing, *n* = 1). The dynamics of conjugates, $\left[ET_{i}\right]$, are given by

(4) $$\frac{dC_{i}}{dt}=k_{1}ET_{i-1}-\left(nk_{2}+k_{-1}\right)C_{i}\;\text{for}\;i=1,\ldots ,n,$$

where *C*_*i*_ values represent the number of $\left[ET_{i}\right]$ conjugates. The corresponding dynamics of the target cells, *T*_*i*_, are

(5) $$\frac{dT_{i}}{dt}=nk_{2}C_{i}+k_{-1}C_{i+1}-k_{1}ET_{i}\;\text{for}\;i=1,\ldots ,n-1.$$

Finally, the rate at which dead target cells appear is

(6) $$\frac{dT^{\text{∗}}}{dt}=nk_{2}C_{n}.$$

We first consider steady-state killing with a fixed total number of target cells, $\bar{T}$, and a fixed number of CTLs, $\bar{E}$. When every target cell, *T*^∗^, that dies is immediately replaced by a fresh target cell, the number of naive target cells, *T*_0_, and the number of free CTLs, *E*, can be solved from the conservation equations as follows:

(7) $$\begin{array}{l} \bar{T}=\sum_{i=1}^{n}C_{i}+\sum_{i=0}^{n-1}T_{i},\\ \bar{E}=E+\sum_{i=1}^{n}C_{i}. \end{array}$$

Thus, $T_{0}=\bar{T}-C_{T}-P$ and $E=\bar{E}-C_{T}$, where $C_{T}=\sum_{i=1}^{n}C_{i}$ is the total number of conjugates, and $P=\sum_{i=1}^{n-1}T_{i}$ is the total number of partially targets.

**Figure 1:**
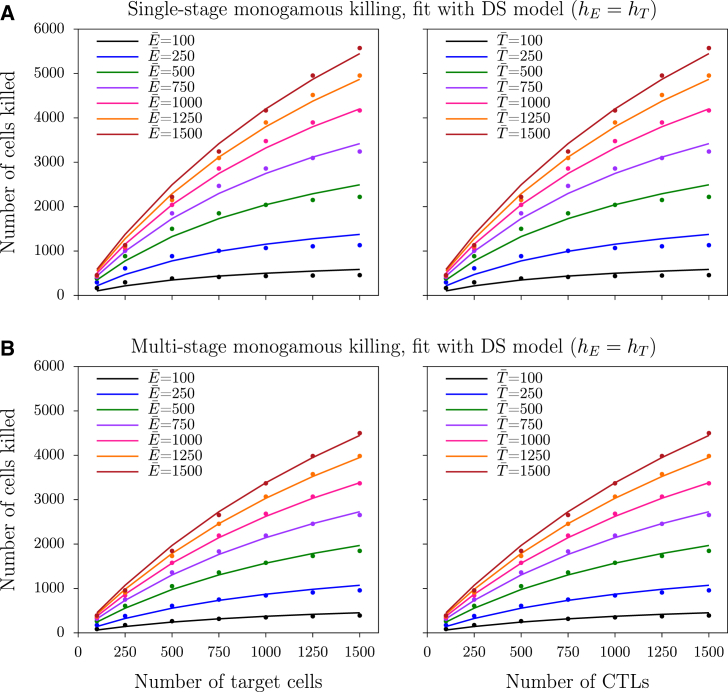
Number of cells killed in the ODE model during the steady state of monogamous killing. (*A* and *B*) Number of cells killed over 75 min during single-stage and multistage (with five stages) killing, respectively. (*Markers*) Observations from the ODE simulations; (*solid lines*) best-fit DS model predictions of Eq. 2 (see Table 3 for the best-fit parameters). To see this figure in color, go online.

To determine the influence of multistage killing on the functional response, we numerically solve the ODEs (Eqs. 4–7), with different numbers of antigen-specific CTLs, $\bar{E}$, and cognate target cells, $\bar{T}$. Thus, we run the model until it approaches steady state, and subsequently measure the number of target cells killed, *T*^∗^, over the next 75 min, for both single-stage killing (setting *n* = 1) and multistage killing (setting *n* = 5). In both scenarios, the number of cells killed saturates to the same extent with an increase in CTL or target cell densities (Fig. 1, *symbols*), which is consistent with the earlier proposed DS functional response (see Eq. 2) for single-stage monogamous killing (1, 4). The DS model describes the single- and multistage killing data reasonably well (except when CTL and target cell densities are high; see the *lines* in Fig. 1). Importantly, the best-fit saturation constants suggest that killing saturates at higher CTL and target cell densities in multistage than in single-stage killing (Table 3).

#### Mechanistic derivation of the functional response for multistage monogamous killing

To better understand why multistage killing results in a saturation of the killing rate at higher cell frequencies than single-stage killing, we next derive the functional response of CTL-mediated killing in the ODE model by extending the total quasi-steady-state approach proposed by Borghans et al. (1) for multistage killing. Thus, similar to our earlier studies (1, 4), we rewrite Eq. 4 in terms of total cell numbers:

(8) $$\begin{array}{l} \frac{dC_{1}}{dt}=k_{1}\overset{\text{free}\;\text{CTLs}\left(E\right)}{\overset{︷}{\left(\bar{E}-C_{T}\right)}}\overset{\text{naïve}\;\text{targets}\left(T_{0}\right)}{\overset{︷}{\left(\bar{T}-P-C_{T}\right)}}-\left(nk_{2}+k_{-1}\right)C_{1},\\ \frac{dC_{2}}{dt}=k_{1}\overset{\text{free}\;\text{CTLs}\left(E\right)}{\overset{︷}{\left(\bar{E}-C_{T}\right)}}\overset{\text{partially}\;\text{lysed}\;\text{targets}}{\overset{︷}{T_{1}}}-\left(nk_{2}+k_{-1}\right)C_{2},\\ \vdots \\ \frac{dC_{n}}{dt}=k_{1}\overset{\text{free}\;\text{CTLs}\left(E\right)}{\overset{︷}{\left(\bar{E}-C_{T}\right)}}\overset{\text{partially}\;\text{lysed}\;\text{targets}}{\overset{︷}{T_{n-1}}}-\left(nk_{2}+k_{-1}\right)C_{n}, \end{array}$$

where $C_{T}=\sum_{i=1}^{n}C_{i}$ is the total number of conjugates, and $P=\sum_{i=1}^{n-1}T_{i}$ is the total number of partially targets. One can sum these equations to obtain the total number of conjugates:

(9) $$\frac{dC_{T}}{dt}=k_{1}\left(\bar{E}-C_{T}\right)\left(\bar{T}-C_{T}\right)-\left(nk_{2}+k_{-1}\right)C_{T}.$$

At steady state, this delivers a quadratic equation

(10) $$C_{T}^{2}-\left(h^{\prime }+\bar{E}+\bar{T}\right)C_{T}+\bar{E}\bar{T}=0,$$

where $h^{\prime }=\left(nk_{2}+k_{-1}\right)/k_{1}$ is the Michaelis-Menten constant.

Next, we observe that at steady state one can add each $dT_{i}/dt$ in Eq. 5 to the corresponding $dC_{i+1}/dt$ from Eq. 4 to obtain that $nk_{2}\left(C_{i}-C_{i+1}\right)=0$, which implies that $C_{i}=C_{i+1}$ for all *i*. Hence, the total number of conjugates is given by $C_{T}=\sum_{i=0}^{n}C_{i}=nC$, where *C* is the number of conjugates at each stage of killing. Eq. 10 therefore simplifies into

(11) $$n^{2}C^{2}-\left(h^{\prime }+\bar{E}+\bar{T}\right)nC+\bar{E}\bar{T}=0,$$

which is similar to what we previously derived for single-stage killing (1, 4). Indeed, setting *n* = 1 we obtain the same quadratic equation as before. Therefore, we also obtain similar expressions for *C* for the full solution, or for the Padé approximation:

(12) $$C_{\text{full}}=\frac{1}{2n}\left(h^{\prime }+\bar{E}+\bar{T}-\sqrt{{\left(h^{\prime }+\bar{E}+\bar{T}\right)}^{2}-4\bar{E}\bar{T}}\right),$$

(13) $$C_{\text{Pade}}=\frac{1}{n}\;\frac{\bar{E}\bar{T}}{h^{\prime }+\bar{E}+\bar{T}}.$$

The rate at which the target cells are killed is given by $dT^{\text{∗}}/dt=nk_{2}C_{n}=nk_{2}C$. Therefore, over a time period Δ*t* the number of target cells killed for either the full solution or for the Padé approximation is given by

(14) $$K_{\text{full}}=\Delta t\;nk_{2}C_{\text{full}}=\Delta t\;\frac{k_{2}}{2}\left(h^{\prime }+\bar{E}+\bar{T}-\sqrt{{\left(h^{\prime }+\bar{E}+\bar{T}\right)}^{2}-4\bar{E}\bar{T}}\right),$$

(15) $$K_{\text{Pade}}=\Delta t\;nk_{2}C_{\text{Pade}}=\Delta t\;\frac{k_{2}\bar{E}\bar{T}}{h^{\prime }+\bar{E}+\bar{T}}\;=\;\Delta t\;\frac{k^{\prime }\bar{E}\bar{T}}{1+\bar{E}/h^{\prime }+\bar{T}/h^{\prime }},$$

where *k*′ = *k*_2_/*h*′ is the mass action killing rate realized at low target and CTL densities (4). Interestingly, because the full solution is identical to the one derived before for single-stage killing (compare Eq. 14 to Eq. 4 in Gadhamsetty et al. (4)), multistage killing at steady state only differs in a quantitative manner from single-stage killing. Specifically, the maximum killing rate is approached at high CTL densities, where $K_{\text{Pade}}\to \Delta t\;k_{2}\bar{T}$, which thus remains independent of the number of stages. However, the definition of the saturation constant, $h^{\prime }=\left(nk_{2}+k_{-1}\right)/k_{1}$, explains why the saturation during multistage killing sets in at higher CTL and target cell densities than during single-stage killing, despite the same maximum killing rate *k*_2_ as in single-stage killing. Moreover, the mass-action killing rate *k*′ does decrease for multistage killing because *k*′ = *k*_2_/*h*′. In conclusion, under steady-state conditions the functional response for multistage killing remains qualitatively the same as the one for single-stage killing. Increasing the number of stages for target cells to be killed results in a lower mass action killing rate and saturation at higher CTL and target cell frequencies, without altering the maximum killing rate.

#### Generalization to the DS model

As in single-stage killing (4), the above Padé approximation (Eq. 15) can be phenomenologically generalized for the four killing scenarios, into the previous general DS model that was already given in Eq. 2, i.e.,

(16) $$K_{\text{DS}}=\Delta t\;\frac{k\bar{E}\bar{T}}{1+\bar{E}/h_{E}+\bar{T}/h_{T}},$$

where *k* remains a mass-action killing rate, and *h*_*E*_ and *h*_*T*_ are saturation constants for increasing CTL and target cell densities. For the simplest case where *h* = *h*_*E*_ = *h*_*T*_ and *k* = *k*_2_/*h*, this DS model reduces back to the Padé solution of Eq. 15. At very high CTL densities, i.e., when $\bar{E}/h_{E}\gg 1+\bar{T}/h_{T}$, the total killing rate becomes independent of the CTL density, and now approaches $kh_{E}\bar{T}$, where *kh*_*E*_ gives the maximum killing rate experienced by a single target cell. At low densities of CTLs and target cells, or when both saturation constants are very large (*h*_*E*_, *h*_*T*_ → ∞), the total killing rate approaches the mass-action term, $k\bar{E}\bar{T}$, showing that the killing rate *k* has the same interpretation as a mass-action killing rate (4). Thus, whenever a fitting procedure leads to parameter estimates where *h*_*E*_ → ∞ and *h*_*T*_ → ∞, one should conclude that the data are well described by a mass-action process. If *h*_*E*_ → ∞ or *h*_*T*_ → ∞, the DS model reduces to

(17) $$K_{\text{DS}|{\text{h}}_{\text{E}}\to \infty }=\Delta t\;\frac{k\bar{E}\bar{T}}{1+\bar{T}/h_{T}}\;\text{and}\;K_{\text{DS}|{\text{h}}_{\text{T}}\to \infty }=\Delta t\;\frac{k\bar{E}\bar{T}}{1+\bar{E}/h_{E}},$$

which only has saturation in target cells, *T*, or effector cells, *E*, respectively. For single-stage killing, we previously showed that simulation data from a mixed killing scenario are well described by symmetric saturation constants, *h*_*T*_ = *h*_*E*_; that simultaneous killing leads to stronger saturation in CTL densities, *h*_*E*_ < *h*_*T*_; and that joint killing leads to stronger saturation in target cell densities, *h*_*T*_ < *h*_*E*_ (4).

#### CPM simulations for multistage killing during steady state

**Figure 2:**
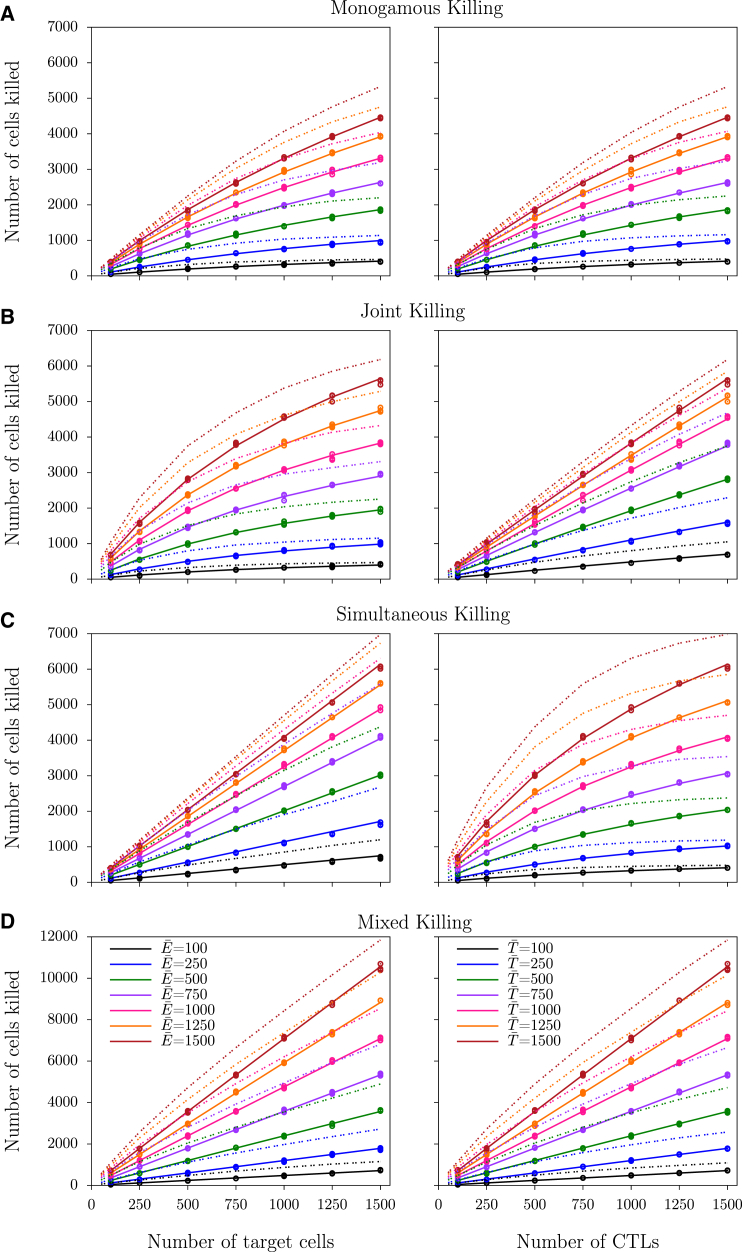
Number of cells killed during the steady state of multistage killing from CPM simulations. (*A*–*D*) Number of cells killed over 75 min during monogamous, joint, simultaneous, and mixed killing, respectively. (*Markers*) Observations from three independent simulations; (*solid lines*) best-fit DS model predictions of Eq. 2 (see Table 3 for the best-fit parameters); (*dotted lines*) average number of cells killed observed in single-stage killing simulations (also shown in Gadhamsetty et al. (4)). To see this figure in color, go online.

An analytical derivation of the functional response was not possible for single-stage joint and mixed killing regimes (4). Moreover, a multistage analytical derivation becomes cumbersome for simultaneous killing. Therefore, we perform CPM simulations in which conjugates of CTL-targets dissociate frequently, to examine whether the above findings can be generalized to all killing regimes. To determine the functional response during steady state, we vary the number of antigen-specific CTLs and cognate targets between simulations. The dissociated target cell retains the killing signal already developed and the killing signal by subsequent conjugates with CTLs accrues on the existing signal, which represents transit through multiple stages to be killed. We measure the number of cells killed over the last 75 min from three independent simulations. Because we measure killing over such a relatively long period, the variation between simulation repeats is very low, which can be appreciated from the almost overlapping markers (see figures described below). First, we perform multistage monogamous killing simulations and find that the number of cells killed saturates with an increase in both CTL and target cell densities (Fig. 2 *A*, *markers*), and the DS model describes the killing well except at high CTL and target cell densities (Fig. 2 *A*, *solid lines*). This is consistent with the results from the ODE model (Fig. 1).

To quantify the saturation during monogamous killing, we fit the DS model of Eq. 2. to the CPM simulation data, and find that a DS model with *h*_*T*_ = *h*_*E*_ describes the data well (Fig. 2, *lines*). In multistage killing, we find that the killing rate (as assessed by the total number of target cells killed over 75 min) saturates at approximately threefold higher CTL and target cell densities compared to single-stage killing (see Table 3 and compare to *dashed lines* in Fig. 2 *A*, which show the simulation results for single-stage killing). Thus, the maximum killing rate during multistage killing is only achieved at very high cell densities, showing that multistage killing is inefficient compared to single-stage killing. Because the mass-action killing rate, *k*, is defined as *k*_2_/*h* for monogamous killing (see Eq. 15), *k* directly depends on the saturation constant. Consistent with this, the best-fit values for *k* are lower during multistage than during single-stage killing.

Next, we examine whether these findings can be generalized to nonmonogamous killing regimes. When performing multistage killing simulations for simultaneous, joint, and mixed killing regimes (see Materials and Methods for a detailed explanation of the scenarios), CTL killing generally saturates with an increase in both CTL and target cell densities. Consistent with our previous study (4), the onset of saturation is asymmetric in simultaneous and joint killing regimes (Fig. 2, *B* and *C*), and in the mixed killing regime the number of cells killed increases almost linearly with an increase in CTL or target cell numbers, i.e., there is no evidence for saturation at these densities (Fig. 2 *D*, *markers*). Best fits of the DS model of Eq. 2 describe the data well (see *solid lines* in Fig. 2), and all saturation constants are higher than those of the corresponding single-stage killing (Table 3 and compare to *dashed lines* in Fig. 2, which show the simulation results for single-stage killing). Taken together, these results demonstrate that the DS model provides an excellent description of the CTL-mediated killing even if multiple hits are required to induce target cell death. Importantly, the influence of multistage killing at steady state can be generalized: the saturation of killing sets in at higher cell densities, and consequently the mass-action killing rate *k* decreases. This is interesting because it may contribute to explaining why there is little evidence for saturation in in vivo data (16).

### Killing before the steady state is established

The functional response of CTL-mediated killing is typically derived by making a QSSA for the number of conjugates (1, 2, 4). A steady-state killing rate is likely to be approached during chronic infections, where the number of infected cells and CTLs remain constant over long periods of time. However, during acute infections or in experiments where fresh targets and CTLs are mixed together, they will initially not be conjugates. The kinetics of binding, dissociation, and killing together determine how rapidly the steady-state number of conjugates is approached and maintained. Moreover, it depends on the dynamics of targets and CTLs (e.g., proliferation, ingress, and egress) themselves whether a quasi-steady state is approached during the course of the experiment or infection. Because the validity of the QSSA under experimental conditions is unclear, we here study the killing activity during the initial phase of ODE and CPM simulations.

#### The functional response in the ODE model for monogamous killing

**Figure 3:**
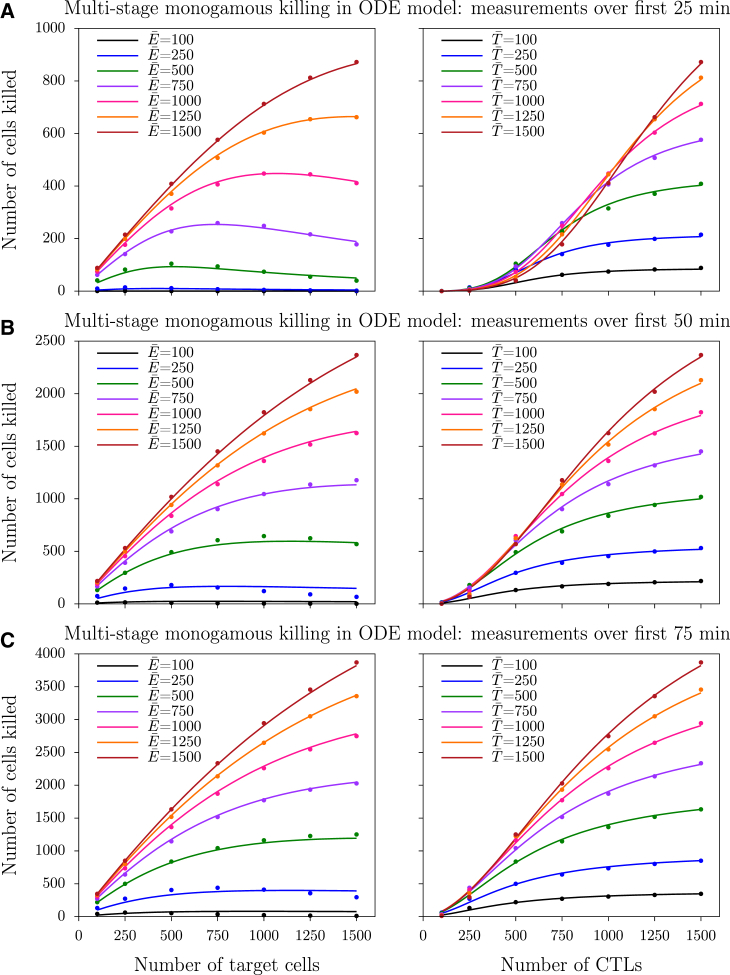
Number of cells killed during the beginning of multistage ODE model simulations. (*A*–*C*) Measurements taken over the first 25, 50, and 75 min, respectively. (*Markers*) Observations from ODE simulations; (*solid lines*) best-fit predictions of the dilution function (Eq. 19). The ODE model was run for 25, 50, or 75 min with a conserved total number of target cells, and with parameters *k*_1_ = 0.001, *k*_−1_ = *k*_2_ = 1/15, and *n* = 5. The best-fit parameters in (*A*) are *k* = 5.16 × 10^−12^, *h*_*E*_ = 574, *h*_*T*_ = 458, *n*_*E*_ = 3.56, and *n*_*T*_ = 2.23; in (*B*) are *k* = 8.14 × 10^−8^, *h*_*E*_ = 422, *h*_*T*_ = 509, *n*_*E*_ = 2.18, and *n*_*T*_ = 1.64; and in (*C*) are *k* = 7.54 × 10^−7^, *h*_*E*_ = 422, *h*_*T*_ = 573, *n*_*E*_ = 1.83, and *n*_*T*_ = 1.49. To see this figure in color, go online.

To determine the functional response before steady state is established, we numerically solve the ODE model for monogamous killing (Eqs. 4–7), now starting with all fresh targets (i.e., $T_{0}=\bar{T}$), for various CTL and target cell densities. As above, fresh targets are replaced when target cells die, i.e., *T*_0_ is solved from the conservation equation (Eq. 7). Data are obtained by counting the number of dead targets, *T*^∗^, that accumulates over the first period of the simulation. For single-stage killing in this model (*n* = 1), the number of cells killed saturates with increases in both CTL and target cell densities in a manner that is qualitatively similar to that observed during steady state (data not shown). In contrast, for multistage killing (employing *n* = 5), the number of cells killed exhibits a sigmoid dependence on the CTL densities (Fig. 3, *markers*). Moreover, an increase in target cells results in a normal saturation at high CTL densities, but in a peak of the killing efficiency at low densities. This peak in the relationship between the number of cells killed and the total number of targets becomes more pronounced at early time points (compare Fig. 3, *A*–*C*), and at high values of the off-rate *k*_−1_ (data not shown).

To also study the more natural situation where target cells are not replaced, we perform the same set of simulations for a model in which the term representing replacement of dead by fresh targets is omitted. As a result, the fresh targets now obey

(18) $$\frac{dT_{0}}{dt}=k_{-1}C_{1}-k_{1}ET_{0}.$$

As before, we set $T_{0}=\bar{T}$ as the initial condition, solve *E* from the conservation equation (Eq. 7), and otherwise use the same set of ODEs (Eqs. 4 and 5). This modification gives very similar results as the case with replacement of dead targets, i.e., a sigmoid dependence on CTL densities and a peak in the dependence on the initial number of targets (Fig. S1 in the Supporting Material). Therefore, we conclude that these dependencies are due to initial transient effects that disappear as soon as steady state is approached.

#### Explaining the dependencies on cell numbers intuitively

The sigmoid relationship between the number of cells killed and the CTL density in the ODE simulations suggests some form of cooperation between the CTLs (although we have not incorporated explicit cooperation in the model). This emerges because at low CTL densities most target cells receive so few hits from the CTLs that they survive after the initial period of 75 min. On the contrary, at high CTL densities many target cells are killed within that time frame because they form sequential kinapses with multiple nearby CTLs. Target cells remember the accumulated killing signal after dissociation from a CTL, and can therefore be killed by subsequent interactions with CTLs. For instance, if the killing of a target were to require two independent contacts with distinct CTLs, one would expect that at low densities the killing rate takes the form of a term with a quadratic dependence on the CTLs, i.e., a *kTE*^2^ term. At high CTL densities, the quadratic dependence disappears because CTLs no longer need to search for targets. Each target then rapidly forms a new conjugate with one of the many CTLs around them and is killed in ∼*t*_*D*_ = 15 min, which leads to saturation at high CTL densities. Overall, this leads to a sigmoid functional response. Because a much smaller fraction of the synapses is expected to break during single-stage killing than during multistage killing, one indeed expects a much stronger sigmoid effect when CTLs form short kinapses (Fig. 3 *A* versus Fig. 3 *B*).

A similar reasoning explains the peak of the killing efficiency at low CTL densities when the target cell density is increased. This happens because at low CTL and high target cell densities the CTLs that break up a conjugate tend to form conjugates with novel targets. Because of this distraction by other targets, on average target cells acquire less killing signal for an excess of targets than for an optimal number of targets. Thus, this is a dilution effect where the presence of other targets decreases the overall rate at which each target is killed. Because conjugates dissociate frequently during multistage killing, increases in the target cell densities tend to dilute the effect of the CTLs whenever the target cells are clearly outnumbering the CTLs. CTLs are then likely to hop from one target cell to another, resulting in many partially lysed target cells and few dead target cells.

In conclusion, the breaking up of conjugates during multistage killing before the steady state is approached leads to two effects that can be intuitively understood. First, the sigmoid dependence on CTLs emerges because multiple consecutive CTLs are required to kill individual target cells. Second, the killing activity of CTLs is diluted among many targets, leading to a peak in the killing efficiency when target densities are increased.

#### Quantifying the sigmoid and the dilution effects

To quantify and compare data with a sigmoid dependence on CTL densities, and a peak in the dependence on target densities, we propose a dilution function, i.e., a phenomenological extension of the DS function:

(19) $$K_{\text{dilution}}=\Delta t\;\frac{k{\bar{E}}^{n_{E}}\bar{T}}{1+{\left(\bar{E}/h_{E}\right)}^{n_{E}}+{\left(\bar{T}/h_{T}\right)}^{n_{T}}},$$

where *h*_*E*_ and *h*_*T*_ remain constants measured in dimensions of CTL and target cell densities, and *n*_*E*_ and *n*_*T*_ are nondimensional exponents allowing for a sigmoid dependence on CTLs and a peak in the dependence on targets (note that *n*_*T*_ is only present in the denominator). The advantage of this function is that for *n*_*E*_ = *n*_*T*_ = 1, the *k*, *h*_*E*_, and *h*_*T*_ parameters keep the mechanistic interpretation of the previous DS function (4), and *n*_*E*_ defines the sigmoid nature in a manner similar to a conventional Hill function. Additionally, one can test various extensions of the DS model in a nested manner, such that variations can be compared by an F-test. For example, the data in Fig. 3 suggest that *n*_*E*_ > 1 to have a sigmoid dependence on CTL, and *n*_*T*_ > 1 to have a peak in the dependence on the target cell densities. Moreover, in general it is expected now that *h*_*E*_ ≠ *h*_*T*_ because *h*_*E*_ is a saturation constant and *h*_*T*_ defines the density of targets where the dilution effect starts to have an impact. Note that the *k* parameter loses its interpretation of a mass action killing rate (with dimension per CTL per time) whenever *n*_*E*_ > 1, whereas *h*_*E*_ and *h*_*T*_ continue to be measured in cell numbers.

Because killing data obtained at early time points reveal dependencies with a sigmoid and peaked nature (Fig. 3), we fit all non-steady-state data with the novel (to our knowledge) dilution function of Eq. 19. Note that steady-state data are expected to lead to parameter estimates where *n*_*E*_ ≃ *n*_*T*_ ≃ 1. However, because the DS function is only an approximation of the full solution (Eq. 14) (4), the exponents might slightly differ from 1, because there is room for the phenomenological dilution function to improve the quality of the fit by slightly changing the shape of the saturation function. Fitting the model to the non-steady-state killing data, we find that both exponents are estimated to be markedly larger than 1 (see legend of Fig. 3 for parameters), and that the function describes the data well (compare *lines* and *markers* in Fig. 3). Very similar results are found when target cells are not replaced (see Fig. S1).

The parameter estimate *n*_*E*_ > 1 confirms that multistage killing leads to sigmoidal functional responses before steady state is approached. Because we did not incorporate such cooperativity in the assumptions underlying the ODE model, this implies that observation of a sigmoid effect in functional response data should not be taken as evidence for cooperation between CTLs. Below, we will estimate *n*_*E*_ at various time points during the approach to steady state. The parameter estimate *n*_*T*_ > 1 confirms that multistage killing leads to a dilution effect, leading to a peak in the killing efficiency when target cell densities are increased. However, in the ODE simulations this dilution effect could be exaggerated because they consider well-mixed CTL and target cells. This implies that a CTL dissociating from a particular target will subsequently bind to any other target with equal likelihood. In real collagen gels and in vivo, CTLs are more likely to bind to nearby target cells, and are hence also more likely to rebind the target that they just dissociated from. We will therefore test our findings in our spatially explicit CPM simulations.

#### Killing during the initial period of spatial simulations

**Figure 4:**
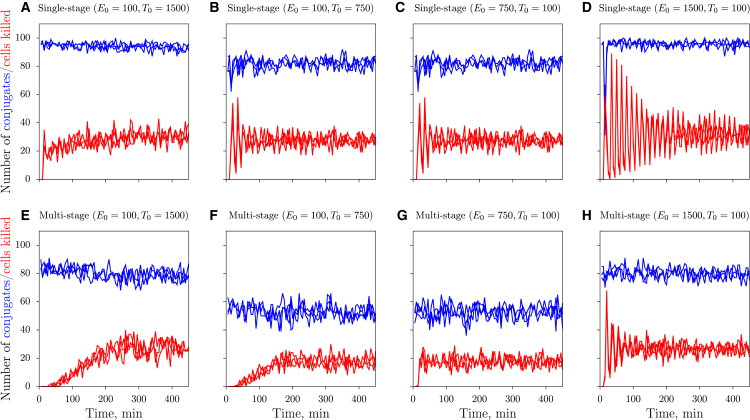
Dynamics of the single- and multistage killing CPM simulations. The total number of synapses (*blue*) and number of cells killed during 5-min time periods (*red*) for monogamous single-stage (*top row*) and multistage (*bottom row*) killing. (*A* and *E*) *E*_0_ = 100 and *T*_0_ = 1500; (*B* and *F*) *E*_0_ = 100 and *T*_0_ = 750; (*C* and *G*) *E*_0_ = 750 and *T*_0_ = 100; and (*D* and *H*) *E*_0_ = 1500 and *T*_0_ = 100 CTL and targets. (*Solid lines*) Observations from three independent simulations. To see this figure in color, go online.

To examine whether the dependencies on cell densities with sigmoid and peaked nature also arise in spatial environments, we perform CPM simulations and measure the number of target cells killed during the initial period of monogamous simulations. Because simulations are starting with fresh target cells, killing does not occur at all during the first *t*_*D*_ = 15 min. In all cases, the total number of CTL-target conjugates rapidly approaches steady state (*blue lines* in Fig. 4), yet the number of cells killed (measured over 5 min intervals) takes a long time to approach steady state (*red lines* in Fig. 4). The approach of the steady-state killing rate is slowest for low CTL densities and for multistage killing (compare *red lines* among the panels in Fig. 4). This is again due to the dilution effect occurring during multistage killing: Because CTLs deliver killing signals to several target cells sequentially, this delays the onset of death of the first target cells as well as the approach to steady state. Thus, the killing signal of a CTL is diluted over several targets, which leads to target cell death only after a period that is typically much longer than the considered killing time (15 min).

Surprisingly, at high CTL, densities dampened oscillations occur with a period of ∼*t*_*D*_ = 15 min (Fig. 4). This is because during single-stage killing all target cells are rapidly bound by one of the CTLs in their neighborhood, die after ∼15 min, and are replaced by fresh in silico targets. These new target cells also rapidly bind one of the many CTLs in their local neighborhood, after which a next wave of dead targets follows. This synchronized killing due to the initial condition only slowly vanishes. The larger the amount of CTLs present, the stronger the oscillatory effect because the search time becomes ultimately negligible (Fig. 4, *A*–*D*). During multistage killing the oscillations are less pronounced because the dilution effect leads to cell death after a more variable period than the rigid 15 min of single-stage killing. However, for multistage killing with an excess of CTLs the oscillations again become strong because targets that lose contact are immediately attacked by another CTL (Fig. 4 *H*). In summary, these observations suggest that the oscillations are due to the same, fixed killing time *t*_*D*_ for all target cells. During in vivo and in vitro studies the killing of target cells will be more asynchronous than in our CPM simulations, because of stochasticity in the killing time and the arrival times of CTLs. Therefore, we performed additional simulations in which the killing times *t*_*D*_ of individual target cells are chosen from a Gaussian distribution (with mean and standard deviation of 15 and 5 min, respectively). As expected, the dampened oscillations are not present in these simulations with a Gaussian killing time distribution (see Fig. S2), demonstrating that they are indeed due to the fixed killing times. Summarizing, the simulations in Fig. 4 highlight that the killing rates may differ considerably between the initial phase of killing and the final steady state.

#### The functional response during the initial period of spatial simulations

**Figure 5:**
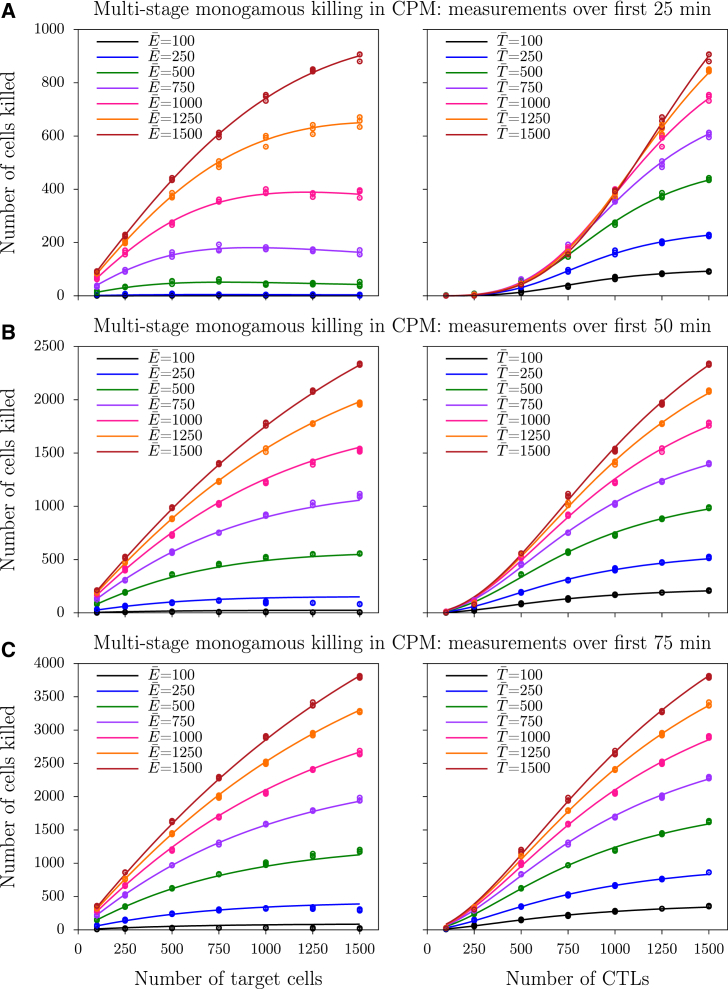
Number of cells killed during the beginning of multistage monogamous CPM simulations. (*A*–*C*) Measurements taken over the first 25, 50, and 75 min, respectively. (*Markers*) Number of cells killed from three independent simulations; (*solid lines*) best-fit predictions of the dilution model (Eq. 19). The best-fit parameters in (*A*) are *k* = 1.99 × 10^−12^, *h*_*E*_ = 854, *h*_*T*_ = 686, *n*_*E*_ = 3.52, and *n*_*T*_ = 1.91; in (*B*) are *k* = 7.72 × 10^−8^, *h*_*E*_ = 707, *h*_*T*_ = 973, *n*_*E*_ = 2.04, and *n*_*T*_ = 1.50; and in (*C*) are *k* = 7.18 × 10^−7^, *h*_*E*_ = 727, *h*_*T*_ = 1069, *n*_*E*_ = 1.71, and *n*_*T*_ = 1.33. To see this figure in color, go online.

To determine the functional response during the initial phase of the simulations, we again vary the antigen-bearing target cell and their cognate CTL densities, but now count the number of cells killed during the initial period of the simulation (25, 50, and 75 min), rather than during the final period of the simulations (i.e., at steady state). In agreement with the data from the ODE model, simulation data from the CPM also suggest a sigmoid relationship between the number of cells killed and the number of CTLs during multistage killing (Fig. 5, *markers*). Importantly, this sigmoid nature becomes less apparent with longer durations of the measurements. Likewise, there is a slight peak in the dependence of the killing efficiency on the number of target cells, which is best visible in the left panel of Fig. 5 *A*. Compared to the ODE simulations (Fig. 3 *B*), the dilution effect seems slightly weaker in the CPM simulations, which can be explained by the higher probability for CTLs to rebind to the target that they just dissociated from in the latter case.

**Figure 6:**
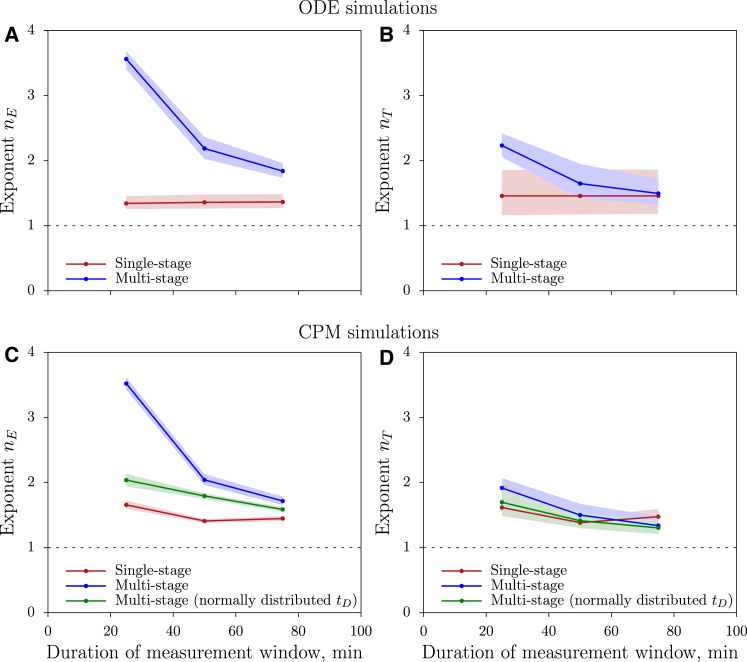
Dependence of the sigmoid and dilution effects on the measurement time window in ODE and CPM simulations. The best-fit exponents *n*_*E*_ (*A* and *C*) and *n*_*T*_ (*B* and *D*) were determined for different measurement periods in the ODE (*A* and *B*) and CPM (*C* and *D*) simulations. (*Markers*) Best estimate for the exponents; (*shaded regions*) their 95% confidence intervals (estimated by bootstrapping the data). The ODE model was run with a conserved total number of target cells, and with parameters *k*_1_ = 0.001, *k*_−1_ = *k*_2_ = 1/15, and *n* = 1 (for single-stage killing; *red*) or *n* = 5 (for multistage killing; *blue*). The CPM simulations were run for single-stage killing with *t*_*D*_ = 15 min (*red*), for multistage killing with *t*_*D*_ = 15 min (*blue*), and for multistage killing with normally distributed *t*_*D*_ (*μ*_*k*_ = 15 min, *σ*_*k*_ = 5 min; *green*). (*Dashed lines*) Expected *n*_*E*_ = 1 and *n*_*T*_ = 1 at steady state. To see this figure in color, go online.

Both the sigmoid and the dilution effect can now be quantified by fitting the dilution function (Eq. 19) to the data, and estimate the exponents, *n*_*E*_ and *n*_*T*_. Above we have shown analytically that the exponent *n*_*E*_ should be close to unity for single- and multistage killing when the system is at steady state. Because this predicts that the exponent should decline for longer measurement periods, we next study how the fitted *n*_*E*_ and *n*_*T*_ depend on the measurement interval and whether this differs for the ODE and CPM simulations. Indeed, these exponents depend on the measurement interval, and get closer to unity when the killing is measured during such long intervals that most of the killing occurs at steady state (see Fig. 6). This occurs because target cells slowly approach a steady-state distribution of accumulated killing signals, implying that a constant fraction of all targets will be killed upon the next encounter with a CTL. Note that *n*_*T*_ is estimated to be higher in the ODE simulations compared to the CPM simulations (compare Fig. 6, *B* and *D*), which is consistent with the expectation that the dilution effect is strongest in the well-mixed case.

Because the CPM simulations with fixed killing times exhibited dampened oscillations in the numbers of cells killed during the initial period of the simulations (Fig. 4), this may affect the sigmoid dependence on CTL numbers and the peak in the dependence on target cell numbers quantitatively. However, we still expect these dependencies to be present in scenarios without oscillations for three reasons. First, for multistage killing the artificial oscillations only occur at very high CTL densities whereas the sigmoid and dilution effect occur already at low cell densities. Second, we count the number of killed cells over periods longer than the cycle time of the oscillations, thus diminishing the impact of the oscillations. Third, we also observed these effects in the ODE simulations that do not exhibit oscillations. Consistent with this reasoning, we obtained qualitatively similar results in the simulations with normally distributed killing times (Fig. S3), although the sigmoid effect does depend quantitatively on the distribution of killing times (Fig. 6 *C*). In conclusion, the spatially explicit CPM simulations confirm that during the initial period of killing, the functional response is sigmoid in CTL density and has a peak in the dependence on target density. Moreover, the best-fit exponent *n*_*E*_ decreases over time, eventually approaching 1 when the killing is measured during such long intervals that most of the killing occurs at steady state.

## Discussion

We have shown that the killing of target cells by the accumulation of several short-lived cytotoxic kinapses at steady state results in a similar functional response as killing by long-lived synapses: a previously defined double saturation function saturating in both CTL and target cell densities (4) describes simulation data from both a CPM and an ODE model well. If the total contact time required for killing is the same in single-stage and multistage killing, the main difference between the two cases is that saturation occurs at lower cell densities in the former case. This qualitative similarity no longer holds for simulation data collected during the initial period of killing when fresh targets are mixed with CTLs. In that case, we find a sigmoid dependence of the killing rate on the CTL density, and a peak in the dependence on the target cell density. These dependencies are due to a dilution effect that can be well described by a phenomenological extension of the DS function with two exponents. When the classical DS model is fitted to killing data with a sigmoid relation between the killing rate and the CTLs, this leads to a compromise in the fit involving a saturation in higher CTL than target cell densities, because of the slow killing rate increase at low CTL densities. Importantly, this can be confused with a joint killing scenario (as in Fig. 2 *B*), which also leads to a difference in the two saturation constants in that direction (4). Therefore, it is important to visually check whether the killing rate has a sigmoid dependence on CTL density, to know if the killing is measured during an initial transient stage or at steady state, and if appropriate to then use the novel (to our knowledge) dilution model we described here.

In enzyme-substrate kinetics, i.e., the analogy that we applied to CTL-mediated killing, cooperativity occurs when the binding of molecules (CTLs, in our case) to a target increase the probability of the binding of subsequent molecules (CTLs), or when the reaction greatly speeds up due to the binding of subsequent molecules (CTLs). It is well known that such cooperativity in enzyme binding results in a more-than-linear increase in the reaction speed with an increase in the enzyme concentration, i.e., leads to a sigmoid dose response (in our case, called “functional” response). Yet in our simulations the sigmoid dependence of the killing rate on CTL densities arises without underlying cooperative mechanisms: conjugates of only a single CTL and a single target cell are allowed to form, and the killing signal is simply added between consecutive conjugates (i.e., a linear increase). For example, one synapse of 15 min has the same effect as two subsequent kinapses of 7.5 min. Additionally, we did not find evidence for a sigmoid dependence in killing data acquired at steady state, even when multiple CTLs bind a single target and kill faster than a single CTL (i.e., during joint killing). Taken together, our results suggest that there are alternative mechanisms, other than cooperation, that result in a sigmoid dependence. Importantly, this implies that observing a sigmoid functional response in killing assays involving kinapses provides no proof for cooperativity, but may simply be a consequence of dilution of the killing signals provided by CTLs. Thus, the recent work demonstrating that CTLs cooperate during the multiple sequential kinapses they form with MCMV-infected cells (5) does show that multiple kinapses with CTLs are required to kill target cells, yet is insufficient to demonstrate true underlying cooperativity.

We considered targets to remember the total duration of time they already spent in conjugation with CTLs when they break a synapse, i.e., during every contact targets advance a killing stage. To our knowledge, there is no experimental evidence supporting or denying this, and touched target cells might be able to recover during the intervals they are not conjugated with a CTL. Although such an infinite memory of the killing signal is the best-case scenario from the perspective of CTLs, our conclusions are independent of that memory, because our extended ODE model simulations with recovery of partially lysed target cells also resulted in qualitatively similar effects (sigmoid and peaked nature; see Fig. S4). Even without memory loss, multistage killing markedly reduces the killing efficiency compared to single-stage killing, and much higher CTL densities are required to obtain the same, maximum killing rate. This situation is even worse during the initial phase of killing fresh target cells because the killing signals that CTLs deliver during short kinapses are distributed over several target cells. It therefore takes a long time before the first target cell dies. Consistent with this, it was recently shown that CXCR3^−/−^ T cells, compared to wild-type T cells, have shorter interactions with virus-infected target cells as well as reduced control of the virus (17). Although the lack of CXCR3 on T cells also affects T cell chemotaxis toward target cells (18) (and thus diminishes killing through less efficient target cell detection), our results suggest that the reduced control could be partly due to less-stable synapses leading to inefficient killing.

The aim of this study was to identify the qualitative nature of the functional response when the killing is not at steady state and/or involves multiple stages, and not to provide quantitative predictions of in vivo killing rates. This allowed us to restrict our analysis to 2D simulations and to avoid three-dimensional (3D) simulations that are prohibitively expensive for such a comprehensive analysis. Nevertheless, our qualitative conclusions are also valid for 3D simulations for two reasons. First, the differences between 2D and 3D killing rates are quantitative rather than qualitative because encounter of targets by CTLs is more efficient in 3D spaces (19, 20). Second, we find similar results in ODE models that consider well-mixed environments of arbitrary dimension. Apart from robustness with respect to dimensionality of the spatial environment in which killing takes place, our results do not depend on variations in the definition of the killing time, as we find similar results in ODEs with exponentially distributed killing times, in CPM simulations with fixed killing times, and in CPM simulations with normally distributed killing times.

Summarizing, our previous DS function (4) can be phenomenologically extended with two parameters allowing for a sigmoid dependence of the killing rate on CTL densities and a peak in the dependence on target cell densities. This extension is probably required to quantify data acquired in the absence of a steady state, and which clearly exhibit a functional response with a sigmoid or peaked nature. Most importantly, we have shown that during initial transients a sigmoid dependence on CTLs can emerge as the mere consequence of sequential killing of a target cell by several CTLs, without having true cooperation between CTLs.

## Author Contributions

S.G., J.B.B., and R.J.d.B. conceived and designed the study, developed the methodology, performed the analysis, and wrote the article; A.F.M.M. developed the simulation environment; and S.G. performed the simulations.

## Figures and Tables

**Table 1:** Summary of Simulation Parameters Used

Parameter	Symbol	Value Used
Time required to kill a target	*t*_*D*_	15 min (15, 21)
Inelasticity of the cells	*λ*	200
Membrane fluctuation amplitude of cells	*D*	6
Directional propensity of CTLs	*μ*_CTL_	450
Directional propensity of targets	*μ*_tgt_	220
Target direction update interval	—	3 min
Number of static elements representing the reticular network	—	1050
Diameter of a reticular network element	—	8 *μ*m
Total number of CTLs	—	2250
Total number of target cells	—	2250
Target area of CTLs and target cells	*A*	44 *μ*m^2^

Parameters are chosen such that the simulated CTLs and B cells recapitulate migration properties observed in vivo. Surface energy parameters are mentioned in Table 2.

**Table 2:** Default Surface Energies and Surface Tensions Used in the Simulations

	ECM	RN	CTL	Tgt
ECM	*J*_ECM,ECM_ = 0	*γ*_ECM,RN_ = 0	*γ*_ECM,CTL_ = 0	*γ*_ECM,tgt_ = 0
RN	*J*_RN,ECM_ = 0	*J*_RN,RN_ = 0	*γ*_RN,CTL_ = 150	*γ*_RN,tgt_ = 150
CTL	*J*_CTL,ECM_ = 150	*J*_CTL,RN_ = 300	*J*_CTL,CTL_ = 300	*γ*_CTL,tgt_ = 0
Tgt	*J*_tgt,ECM_ = 150	*J*_tgt,RN_ = 300	*J*_tgt,CTL_ = 300	*J*_tgt,tgt_ = 300

Surface energies are represented by *J*, and surface tensions by *γ*. Upon conjugate formation, we decrease the surface tension between CTL-target pairs with empirically chosen adhesion strength: by *γ*_adhesion_ = 60 for single-stage killing, and by *γ*_adhesion_ = 5 for multistage killing. Thus, the effective surface tension between CTLs and targets in a conjugate becomes *γ*_CTL,tgt_ − *γ*_adhesion_. ECM, extracellular matrix; RN, reticular network; Tgt, tgt, target cell.

**Table 3:** Summary of Best-Fit Parameters of the DS Model (Eq. 2) and the Corresponding 95% Confidence Intervals

Parameter	*k* (×10^5^)	*h*_*E*_	*h*_*T*_
Dimension	cells^−1^ min^−1^	cells	cells

Single-stage killing			

Monogamous (ODE)	17.3 (13.4–21.4)	*h*_*E*_ = *h*_*T*_ = 682 (477–887)	
Monogamous	18 (16.6–19.4)	*h*_*E*_ = *h*_*T*_ = 571 (503–639)	
Simultaneous	18 (9.9–26.1)	523 (487–559)	2979 (2418–3540)
Joint	18 (14.0–22.0)	2051 (1907–2195)	458 (443–473)
Mixed	17 (16.5–17.5)	*h*_E_ = *h*_T_ = 1945 (1814–2076)	

Multistage killing			

Monogamous (ODE)	2.0 (1.1–9.2)	*h*_E_ = *h*_T_ = 1047 (892–1202)	
Monogamous	6.7 (6.5–6.8)	*h*_E_ = *h*_T_ = 1950 (1884–2018)	
Simultaneous	7.1 (6.9–7.1)	1602 (1560–1644)	∞
Joint	7.4 (7.2–7.5)	12,792 (9922–15,661)	1364 (1314–1415)
Mixed	6.5 (6.4–6.6)	*h*_E_ = *h*_T_ = 70,385 (47,172–93,598)	

Confidence intervals are given in parentheses in columns 2–4 (based on asymptotic normal distributions for the parameters) for single-stage and multistage killing under steady-state conditions, obtained from nonspatial dynamical (marked with *ODE* in column 1) and CPM simulation models. The best-fit parameters for single-stage killing in CPM simulations are taken from our previous study (4).

## Supporting Material

Document S1. Figs. S1–S4

Document S2. Article plus Supporting Material
