# Supplementary material for: A Sigmoid Functional Response Emerges When Cytotoxic T Lymphocytes Start Killing Fresh Target Cells
Source: Biophys J. 2017 Mar 28;112(6):1221–35. doi: 10.1016/j.bpj.2017.02.008 (PMC5375173; doi:10.1016/j.bpj.2017.02.008)
Supplement: Document S1. Figs. S1–S4 [file mmc1.pdf]

Biophysical Journal, Volume *112*

Supplemental Information

A Sigmoid Functional Response Emerges When Cytotoxic T Lymphocytes Start Killing Fresh Target Cells

Saikrishna Gadhamsetty, Athanasius F.M. Marée, Joost B. Beltman, and Rob J. de Boer

Supplementary Information

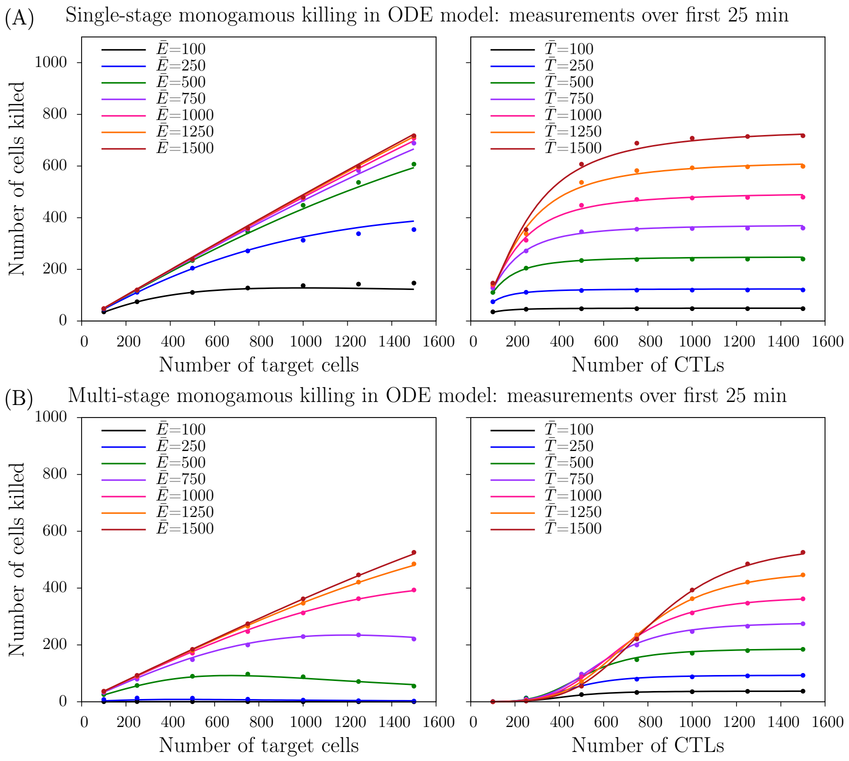

Figure S.1: **Number of cells killed in ODE simulations without replacing dead targets with fresh ones.** Panels (A) and (B) depict the number of cells killed over the first 25 min during single- and multi-stage monogamous killing (with 5 stages). Parameters of the ODE model as in Fig. 3. The observations from the simulations are shown in markers, and the best fits of Eq. 19 are depicted in solid lines. The parameter estimates for single-stage killing are $k = 1.01 \times 10^{-5}$, $h_E = 60.4$, $h_T = 304.9$, $n_E = 1.85$, and $n_T = 1.55$, and for multi-stage killing $k = 8.34 \times 10^{-14}$, $h_E = 436$, $h_T = 493$, $n_E = 4.26$, and $n_T = 2.36$.

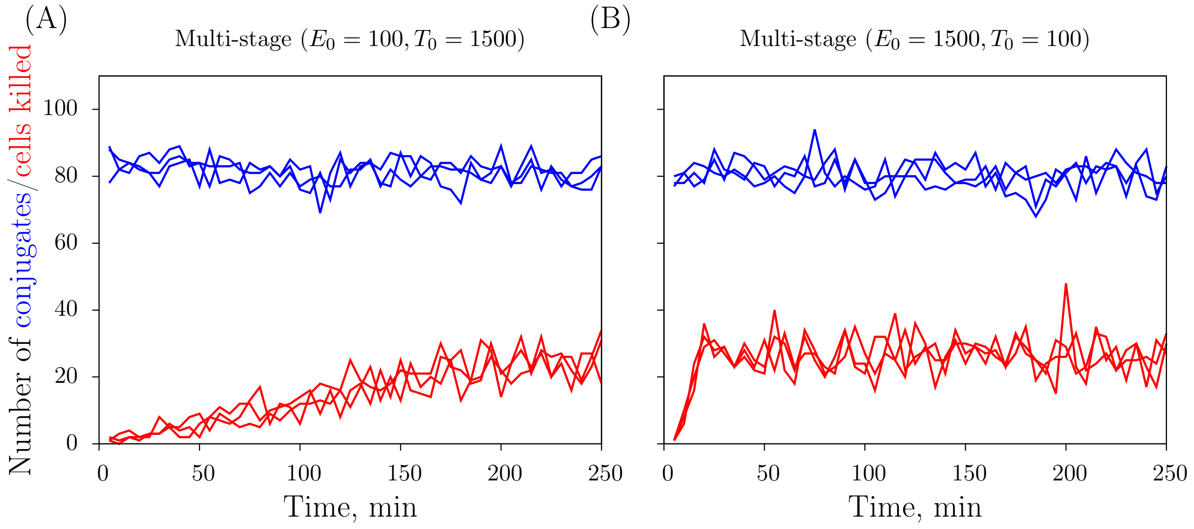

Figure S.2: **Dynamics of the multi-stage killing CPM simulations with killing times sampled from a normal distribution.** The total number of synapses (blue) and number of cells killed during 5-min time periods (red) for monogamous killing. The solid lines represent the observations three independent simulations.

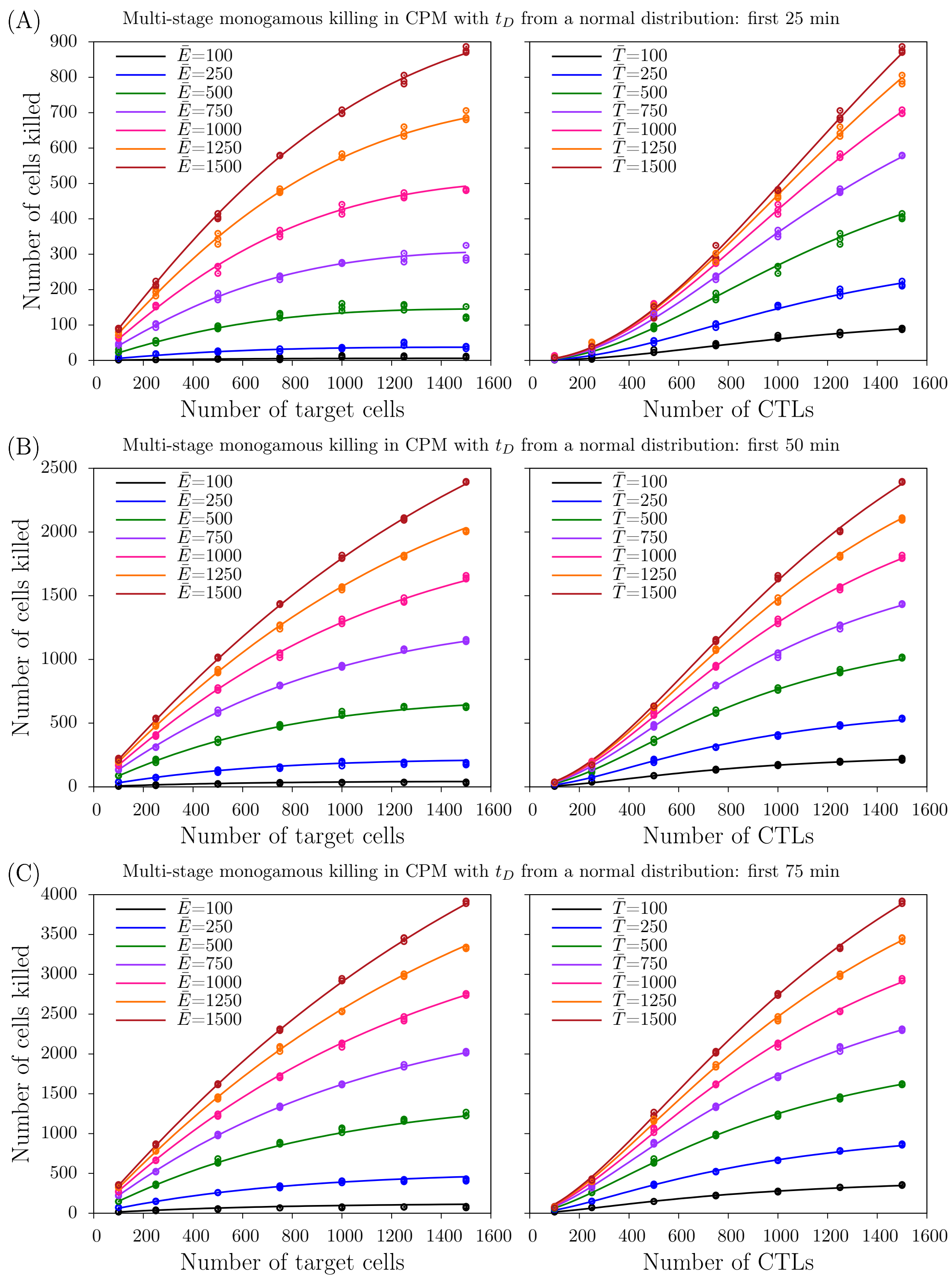

Figure S.3: **Number of cells killed in multi-stage monogamous CPM simulations with a normally distributed killing time.** t_D has a mean $\mu_k = 15$ min and standard deviation $\sigma_k = 5$ min. The number of cells killed obtained from three independent simulations are shown in markers, and the best-fit predictions of the dilution function are depicted in solid lines. The estimates for measurements over the first 25 min are $k = 3.28 \times 10^{-8}$, $h_E = 1151$, $h_T = 1181$, $n_E = 2.03$, and $n_T = 1.69$; over the first 50 min are $k = 3.71 \times 10^{-7}$, $h_E = 774$, $h_T = 1101$, $n_E = 1.79$, and $n_T = 1.41$; and over the first 75 min are $k = 1.59 \times 10^{-6}$, $h_E = 800$, $h_T = 1207$, $n_E = 1.58$, and $n_T = 1.30$.

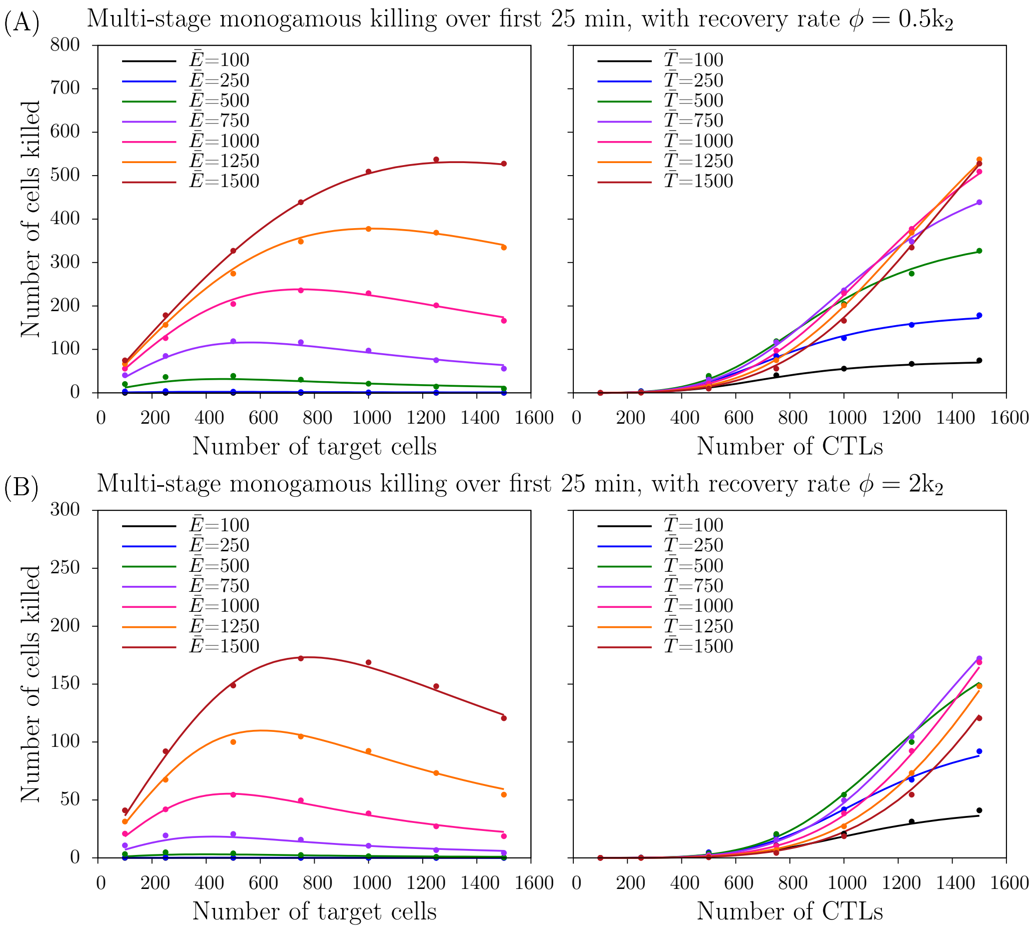

Figure S.4: **Number of cells killed in multi-stage killing monogamous simulations with recovery of partially-lysed target cells.** If partially-lysed target cells recover and go back to the earlier stage of killing at a rate ϕ, the target cell dynamics are given by $\frac{dT_i}{dt} = nk_2C_i + k_{-1}C_{i+1} - k_1ET_i - \phi T_i + \phi T_{i+1}$. The above equation along with Eqs. 4 and 6 are solved starting with fresh target cells, and the number of cells killed over the first 25 mins is determined for $\phi = 0.5k_2 = 1/30$ (i.e., $\phi \ll k_2$; panel (A)) and $\phi = 2k_2 = 1/7.5$ (i.e., $\phi \gg k_2$; panel (B)). Markers represent the measurements from the simulations, and the solid lines represent the best-fit predictions of the DS sigmoid model. The best-fit parameters for $\phi = 0.5k_2$ are $k = 2.11 \times 10^{-13}$, $h_E = 763$, $h_T = 478$, $n_E = 3.86$, and $n_T = 2.34$; and for $\phi = 2k_2$ are $k = 1.63 \times 10^{-16}$, $h_E = 1067$, $h_T = 466$, $n_E = 4.63$, and $n_T = 2.56$.
